# Prospective association between the gut microbiome and incident hypertension: a 20-year cohort study

**DOI:** 10.1097/HJH.0000000000004254

**Published:** 2026-02-09

**Authors:** Li-Fang Yeo, Joonatan Palmu, Aki S. Havulinna, Katariina Pärnänen, Veikko Salomaa, Leo Lahti, Rob Knight, Teemu Niiranen

**Affiliations:** a Division of Medicine, Turku University Hospital; b Department of Internal Medicine, University of Turku, Turku; c Department of Computing, University of Turku, Turku; d Clinical and Molecular Metabolism (CAMM); e Department of Microbiology, University of Helsinki; f Department of Public Health, Finnish Institute for Health and Welfare, Helsinki; g Department of Pediatrics; h Center for Microbiome Innovation, Joan and Irwin Jacobs School of Engineering; i Department of Bioengineering; j Department of Computer Science and Engineering; k Halicioğlu Data Science Institute, University of California, San Diego, La Jolla, California, USA

**Keywords:** gut microbiome, hypertension, prospective study

## Abstract

**Introduction:**

Hypertension remains the leading modifiable risk factor attributable to 10.8 million premature deaths. Hence the study of hypertension and gut microbiome as a therapeutic target is very important. Yet the links between the gut microbiome and long-term incidence of hypertension are unknown.

**Aim:**

This study assessed the association between gut microbiome and incident hypertension.

**Method:**

The study sample consisted of 3311 nonhypertensive individuals (60.7% women) aged 25–74  years who were drawn from the general population in Finland. In the baseline examination performed in the year 2002, the participants underwent a health examination and provided a stool sample. The gut microbiome was assessed using shallow shotgun metagenomic sequencing. Microbiome analyses were performed with Cox proportional hazards model.

**Results:**

In total, 675 participants developed hypertension over a follow-up period of nearly 20 years. In multivariable-adjusted models, overall gut microbiome composition was not related to risk of future hypertension. Eight genera, including *Agathobaculum, Blautia_A_141780, Blautia_A_141781, Mediterraneibacter_A_155590, Enterocloster*, *Bariatricus*, *CAG-317–146760*, and *CAG-628* were significantly associated with incident hypertension in the age-adjusted and sex-adjusted models, but none remained significant in the multivariable-adjusted models. No functional pathways were associated with hypertension risk.

**Conclusion:**

Our results do not provide strong evidence for an association between the gut microbiome and risk of future hypertension, especially after adjusting for covariates that are known to influence the gut microbiome.

## INTRODUCTION

Cardiometabolic diseases (CMD) are the leading cause of death globally. CMD represents 32% of deaths of which 85% were due to heart attack and stroke [1]. The latest Global Burden of Cardiovascular Diseases and Risk 2022 study reported that ischemic heart disease, ischemic stroke, and hypertensive heart disease remain the leading cause of death globally. Hypertension is the leading modifiable risk factor attributable to 10.8 million premature deaths [2]. In Finland, approximately two million adult Finns have elevated blood pressure, which translates to approximately only one in five adults having ideal blood pressure levels [3]. Hypertension screening is highly effective in Finland as approximately 85% of Finns aged 20–75+ years reported having their blood pressure measured in the past 12 months [4].

The gut microbiome has been extensively studied in association with cardiometabolic diseases. For example, the influence of bacteria has long been suggested to contribute to inflammation in atherogenesis and subsequently to coronary artery disease [5]. An interesting study characterized the microbiome and metabolome of individuals ranging from healthy, having only metabolic syndrome but without ischemic heart disease, to people with ischemic heart disease, and found that major alterations in the gut microbiome and metabolome may be detected long before a clinical onset of ischemic heart disease [6]. Furthermore, there have been evidence of cardiac dysfunction aggravated by gut microbes and microbe-mediated metabolites through the gut–kidney–heart axis [7,8]. These studies suggest a pathophysiology mechanism where gut microbes and metabolites are involved in the process of cardiac dysfunction over a long period of time that eventually leads to a cardiac event.

In the case of hypertension, evidence from epidemiological, clinical, and animal studies suggests that gut microbiome dysbiosis is associated with increased odds of hypertension [9,10]. Several reviews in this domain have summarized how blood pressure is regulated by gut microbes, the effect of microbe-derived metabolites on hypertension, and the overall interactions between the gut microbiome and host metabolism and immune system [11,12]. However, most previous studies on the links between the gut microbiome and hypertension have used small cross-sectional study samples [13]. In a meta-analysis conducted by Cai *et al.* in 2023, we can clearly see several studies with less than 100 study participants. These studies are still valuable yet caution must be exercised when drawing conclusions from under-powered studies [14].

Furthermore, cross-sectional studies are subjected to selection bias as only individuals who have developed the disease, or in this case, have hypertension are investigated [15]. As a diagnosis of hypertension usually affects the patient's dietary habits and medication, it is challenging to control for these factors after the diagnosis has been made without further introducing bias into the study [15,16]. Hence, the use of prospective cohort studies, where healthy participants are prospectively followed until overt disease develops could provide a higher level of evidence towards causality than cross-sectional case–control studies [17].

In this study, we assessed the association between the gut microbiome and incident hypertension in a large, prospective Finnish cohort of 3311 normotensive individuals who were followed up for incident hypertension over a median of 19.8 years.

## METHODS

### Study sample and study flow

The FINRISK studies are population-based health examination surveys that have been carried out quinquennially in Finland since 1972. The FINRISK 2002 cohort was a stratified random sample of the Finnish population aged between 25 and 74  years drawn from the national population register in 2002 [18]. Participants were randomly drawn from the national population register from six geographic regions in Finland. In the year 2002, the Finnish population was more than 99% white. We did not collect data on race or ethnicity. Before the baseline examination, the participants filled in a questionnaire on demographics, health-related behaviours, diet, and cardiovascular and general health. Participants then underwent a physical examination at a local study site, where height, weight, and blood pressure were recorded. Blood samples were collected after a 4-h fasting period. At the end of the baseline examination, the participants received a stool collection kit for faecal sampling.

**FIGURE 1:**
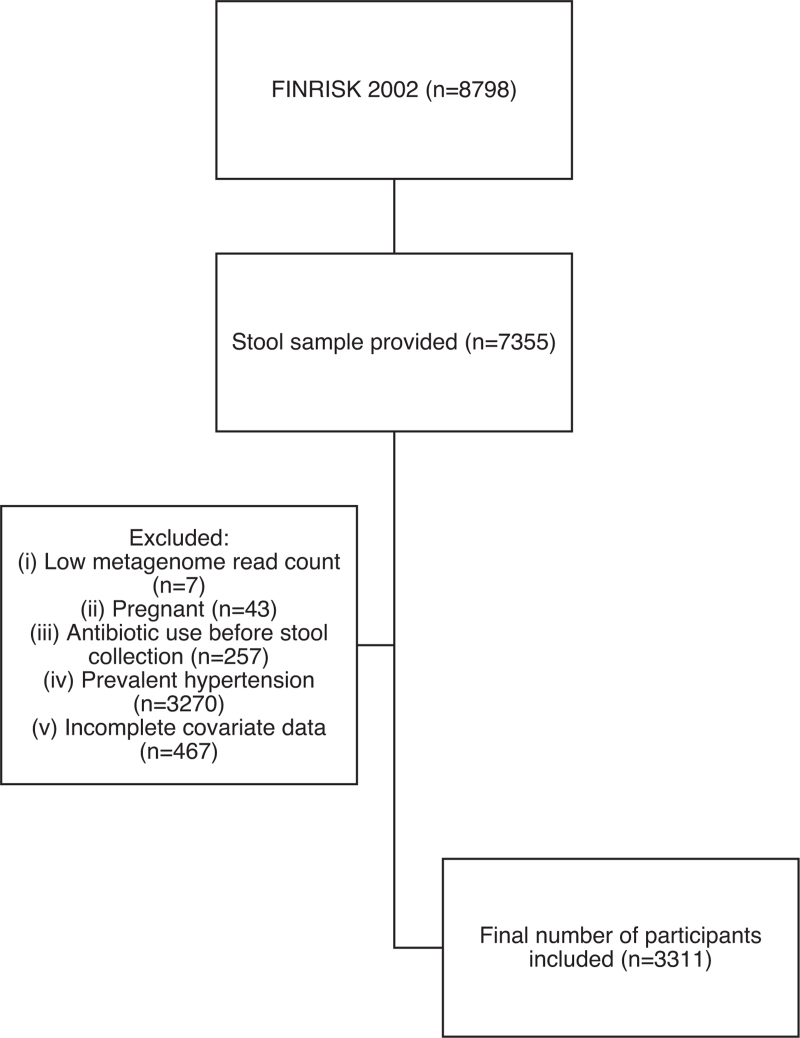
Flow chart of participant inclusion.

The original number of invited participants was 13 500, and 8798 individuals participated in the health examination (overall participation rate 65.5%). A total of 7355 (80.2%) provided a stool sample for microbiome analysis. The exclusion criteria were individuals with a low (*N* < 50 000) metagenome read count (*n* = 7); pregnancy at the time of baseline investigation (*n* = 43); antibiotic use 1 month before stool sample collection (*n* = 257); participants with prevalent hypertension at baseline (*n* = 3263); and incomplete covariate data (*n* = 474). Thus, a total of 3311 normotensive individuals at baseline were included in the analysis. The participants were followed up for incident hypertension using nationwide register data until 31 December 2022 (Fig. 1).

The study has been approved by the Coordinating Ethical Committee of the Helsinki and Uusimaa Hospital District (decision number 87/2001). All participants have signed an informed consent.

### Microbiome sampling and data processing

Stool samples were collected in 50 ml Falcon tubes and mailed overnight to the Finnish Institute for Health and Welfare during the winter months [19]. The samples were frozen in −20 °C until 2017. DNA was extracted, and shallow shotgun metagenomics sequencing was performed at University of California San Diego using a standard Earth Microbiome Project protocol [20]. Samples were sequenced on Illumina Hi-Seq 4000 at 2 × 150 bp read length. Sequences were then mapped to the Greengenes2 database [21]. Functional pathways were annotated using MetaCyc database through the HUMAnN (v 3.0.1) pipeline [22,23]. The data was imported in R as a TreeSummarizedExperiment container, and processed using the mia R/Bioconductor framework [24–26].

### Outcome variable

Incident hypertension was defined as having a hypertension-related diagnosis code in the Hospital Discharge or Causes-of-Death registers [27]. In addition, incident hypertension was defined as having a special reimbursement code for hypertension or a minimum of three drug purchases with the Anatomical Therapeutic Chemical codes for dihydropyridine calcium-channel blockers (CCBs) or thiazide diuretics in the Drug Reimbursement Register (Supplementary Table S1). CCBs or thiazide diuretics drug purchases was chosen as indicators for hypertension as unlike other medications used for treating hypertension, these two groups of pharmaceuticals are almost solely used to treat hypertension.

### Variable definitions

A nurse measured blood pressure three times after a 5-min rest in sitting position using a standard mercury sphygmomanometer with a 1-min rest between the measurements. Blood pressure was considered as the mean of the three measurements. Prevalent hypertension was defined as a SBP at least 140 mmHg, a DBP at least 90 mmHg, self-reported use of antihypertensive medication, or a register-based diagnosis of hypertension prior to baseline (see ‘Outcome variable’ for definition of register-based hypertension).

BMI was defined as weight (kg) divided by the square of height (m). Smoking was self-reported and categorized into two groups: current smokers; and nonsmokers if individuals did not smoke in the past 6 months. Healthy food choices (HFC) were defined as a composite score from the sum of a Food Propensity Questionnaire (FPQ) responses to food items that ranges from 9 to 745, whereby higher scores indicate higher number of healthy food choices made per month [28]. Prevalent cardiovascular disease and diabetes type 1 and 2 were defined using data from nationwide Hospital Discharge Register, and Drug Purchase and Reimbursement Register (Supplementary Table S1). Cardiovascular diagnoses in these registers have been validated and shown to have good coverage and accuracy [27,29].

### Statistical analysis

Alpha diversity measures can be seen as a summary statistic of diversity in a single group (within-group diversity, i.e. participants with incident hypertension). Alpha diversity was calculated at species level using Shannon and observed richness index. Higher alpha diversity has been in some cases associated with improved health outcomes [30]. Beta diversity measures dissimilarity of microbial communities between individual samples. This can be used to measure differences in community composition between participants with and without hypertension. We used the standard Bray-Curtis dissimilarity index at the species level with relative abundance. Beta diversity quantifies community-level (multivariate) differences. This can be further complemented by differential abundance analysis, which quantifies (univariate) differences in individual taxa. Differential abundance analyses were performed at the family, genus, and species levels. To reduce noise, mitigate technical variability, and improve the statistical power of downstream analyses, microbial taxa were filtered in differential abundance analyses for presence in at least 5% of the population at a relative abundance of more than 0.1% [31].Microbial abundance were centred log-ratio (CLR) transformed to account for sparsity and compositional nature of microbiome data.

The relationship between species composition and incident hypertension was visualized with Principal Coordinate Analysis (PCoA).We measured differences in species-level community composition using PERMANOVA with 999 permutations (Bray–Curtis index) [32]. Permutational Multivariate Analysis of Variance (PERMANOVA) compares differences between two groups by first calculating the dissimilarity between each sample and then comparing the centroids of each group for differences [32]. In this case, PERMANOVA assessed differences in microbial community composition of individuals with and without incident hypertension, and quantified what fraction each covariate explained differences in the microbiome composition [33]. Analysis of the functions predicted based on metagenome composition focused on pathways that were assigned to a species and were prevalent in at least 5% of the population. We did not filter by abundance to capture more pathways, as the samples were shallow sequenced where many pathways may be underrepresented. After applying the above filters, the total number of functional pathways included in the analysis was 1515. Due to the high sparsity of the pathway abundance data, we performed the analysis by dichotomizing the pathway abundance (present versus absent), or normalizing functional pathways using inverse rank transformation.

We studied the association between incident hypertension and alpha diversity using Cox proportional hazards model. Analysis of compositions of microbiomes with bias correction 2 (ANCOM-BC2) is a statistical method to identify taxa that has different abundance for the outcome of interest, that is incident hypertension, compared to normotensive participants. In this study, cox proportional hazards model and ANCOM-BC2 with default settings were used to investigate the links between microbial taxa-associated and incident hypertension [33]. Despite ANCOM-BC2 not being designed for time-to-event analyses, we wanted to examine if there were overlaps in significant taxa found in the Cox models. The associations between pathway abundance and incident hypertension were conducted using Cox proportional hazards models.

All analyses were adjusted for age and sex or age, sex, BMI, diabetes type 1 and 2, healthy food choices, cardiovascular disease, and smoking. A sensitivity analysis was conducted by removing BMI from the alpha diversity analysis to determine the confounding effect BMI may have on the model. *P* values in differential abundance analyses and functional pathway analysis were corrected for multiple testing using False Discovery Rate (FDR, Benjamini–Hochberg correction). Cox proportional hazards model assumptions were tested using scaled Schoenfeld residuals. Two-sided *P* values less than 0.05 were considered statistically significant. Analyses were conducted using R version 4.3.3 and mainly using mia package 1.15.17 [25].

### Code availability statement

The source code and full list of R packages used for the analyses and figures are available at https://doi.org/10.5281/zenodo.15773611.

## RESULTS

The characteristics of the study population are summarized in Table 1. The mean age of study participants was 43.4 ± 11.9 years, and 60% were women. Over a median follow-up of 19.8 years, 675 participants developed hypertension. We performed a sensitivity analysis to check for selection bias, as we had to exclude a number of participants with missing covariates. We observed no differences in the baseline characteristics between included and excluded participants, with the exception for DBP (*P* = 0.04, Supplemental Table S2).

**TABLE 1:** Baseline characteristics of the study sample

		Incident hypertension		
Characteristic	Overall	Yes	No	*P*
*N*	3311	675	2636	–
Age (years) (95% CI)	43.4 (43.0–43.8)	47.3 (46.4–48.2)	42.4 (42.0–42.8)	<0.001
Women [*n* (%)]	1986 (60.0%)	394 (58.4%)	1592 (60.4%)	–
BMI (95% CI)	25.6 (25.5–25.7)	27.4 (27.1–27.7)	25.1 (25.0–25.2)	<0.001
Diabetes [*n* (%)]	80 (2.4%)	25 (3.7%)	55 (2.1%)	
SBP (mmHg) (95% CI)	122.0 (121.7–122.3)	126.0 (125.3–126.7)	121.0 (120.6–121.4)	<0.001
DBP (mmHg) (95% CI)	73.6 (73.3–73.9)	76.5 (75.9–77.1)	72.9 (72.6–73.2)	<0.001
Healthy food choices^a^ (95% CI)	191.0 (188.1–193.9)	187.0 (180.1–193.9)	192.0 (188.8–195.2)	0.21
Cardiovascular disease [*n* (%)]	63 (1.9%)	21 (3.1%)	42 (1.6%)	–
Smoking [*n* (%)]	876 (26.5%)	202 (29.9%)	674 (25.6%)	–
Shannon diversity (95% CI)	4.12 (4.11–4.13)	4.11 (4.08–4.14)	4.13 (4.11–4.15)	0.38
Observed richness (95% CI)	1060 (1053–1067)	1080 (1064–1096)	1060 (1052–1068)	0.04

CI, confidence interval.

a Higher healthy food choices indicate more healthy food choices made per month.

*P*-value calculated by Chi-square test.

### Alpha and beta diversities

**FIGURE 2:**
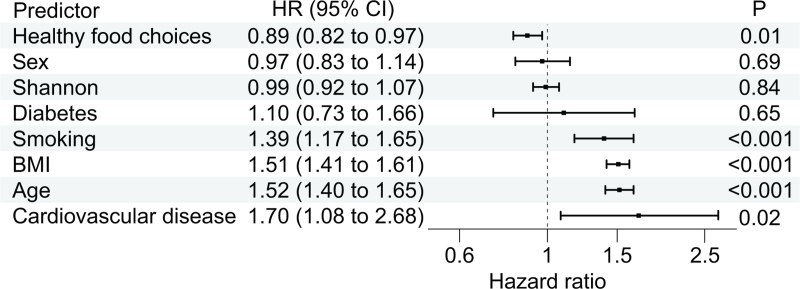
The multivariable-adjusted association between alpha diversity (Shannon index) and incident hypertension.

In age-adjusted and sex-adjusted models, alpha diversity (Shannon) was not associated with incident hypertension [hazard ratio, 0.93; 95% confidence interval (CI), 0.87–1.00; *P* = 0.05; two-tailed Wald test for Cox regression coefficient]. The results were similar in the multivariable-adjusted model (Fig. 2; hazard ratio, 0.99; 95% CI, 0.92–1.07; *P* = 0.84). Observed richness was also not associated with incident hypertension in both models (results not shown).

**FIGURE 3:**
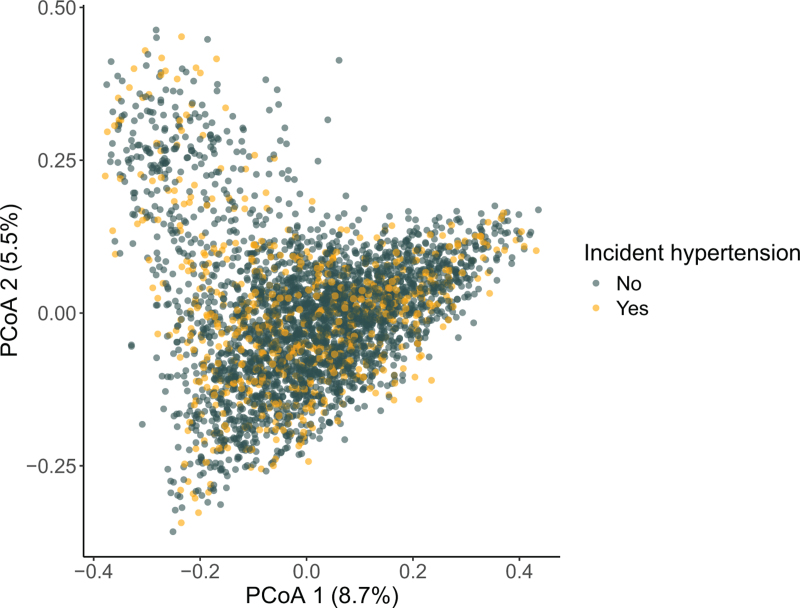
Principal Coordinate Analysis plot showing the dissimilarity in microbiome composition between participants with and without incident hypertension.

Community dissimilarity was visualized on PCoA. Microbiome composition of individuals who developed incident hypertension were similar to those who did not (Fig. 3**)**. We used PERMANOVA to assess the importance of each covariate, including beta diversity, on risk of incident hypertension. Incident hypertension was significantly associated with beta diversity (*P* = 0.03; *F*-statistic; age-adjusted and sex-adjusted), but the model explained less than 0.1% of the variance in gut microbiome species composition. The multivariable-adjusted model explained 2% of the variance, but incident hypertension did not significantly contribute to the model (*P* = 0.56, Supplementary Table S3). These results suggest that beta diversity is not associated with incident hypertension.

### Differential abundance analysis

After prevalence filtering to remove rare taxa and reduce data sparsity, 258 species, 171 genera, and 49 families remained for analysis. In age-adjusted and sex-adjusted Cox models, eight microbial genera, namely *Agathobaculum, Blautia_A_141780, Blautia_A_141781, Mediterraneibacter_A_155590, Enterocloster*, *Bariatricus*, *CAG-317–146760*, and *CAG-628* were significantly associated with incident hypertension (FDR < 0.03 for all; two-tailed Wald test for Cox regression coefficient; Table 2). However, these associations were nonsignificant in the multivariable-adjusted models (FDR ≥ 0.39 for all; Table 2). At the family level, *Alkalibacilaceae*, *Anaerovoracaceae*, *Butyricicoccaceae*, *CAG-138*, *Eggerthellaceae*, and *UBA660* were significantly associated with incident hypertension in the age-adjusted and sex-adjusted models (FDR = 0.04 for all, Supplemental Table S4), but not in the multivariable-adjusted models (FDR ≥ 0.63 for all). Similar results were observed in the species-level analyses, as the results were nonsignificant after adjusting for multiple variables (FDR ≥ 0.07 for all; Supplemental Table S5). As a sensitivity analysis, we also examined the differential abundance between individuals with and without incident hypertension using ANCOM-BC2. In this analysis, a lower relative abundance of the genus *CAG-628* was associated with incident hypertension in the age-adjusted and sex-adjusted models (Supplemental Table S6; log-fold change, −0.34; SE, 0.10; FDR = 0.04; two-sided *Z* test using the test statistic W (log-fold change divided by standard error). This result was robust as it passed the sensitivity analysis, a default setting in ANCOM-BC2 to test for false positives due to the addition of pseudocounts [33]. ANCOM-BC2 analysis at the species and family level did not yield any significant results (Supplemental Tables S7 and S8).

**TABLE 2:** The associations between microbial genera and incident hypertension in Cox proportional hazards models

Genus	HR	95% CI	FDR
Age-adjusted and sex-adjusted model			
*Agathobaculum*	1.23	1.10–1.37	0.02
*Blautia_A_141780*	1.21	1.09–1.35	0.02
*Blautia_A_141781*	1.20	1.09–1.32	0.02
*CAG_317_146760*	1.27	1.11–1.45	0.02
*Mediterraneibacter_A_155590*	1.28	1.12–1.48	0.02
*CAG_628*	0.94	0.90–0.97	0.02
*Enterocloster*	1.14	1.06–1.24	0.02
*Bariatricus*	1.17	1.06–1.30	0.03
Multivariable- adjusted model			
*Eubacterium_G*	0.87	0.8.–0.95	0.39
*Merdousia*	1.05	1.02–1.09	0.39
*Allobacillus*	1.06	1.01–1.12	0.69
*Anaerostipes*	1.08	1.01–1.16	0.69
*Ruminococcus_C_58660*	0.87	0.78–0.98	0.69
*Senegalimassilia*	1.05	1.01–1.09	0.69
*Ellagibacter*	1.03	1.00–1.06	0.78
*Gabonibacter*	1.06	1.00–1.12	0.78
*Intestinimonas*	0.89	0.80–1.00	0.78
*Massilistercora*	1.13	1.00–1.27	0.78

HR, hazard ratio; CI, confidence interval; FDR, false discovery rate corrected *P* for two-tailed Wald test for Cox regression coefficient. Multivariable-adjusted models were adjusted for age, sex, BMI, diabetes, healthy food choices, cardiovascular disease, and smoking.

### Pathway analysis

One unintegrated pathway linked to *Eubacterium hallii* was significantly associated with incident hypertension after dichotomization of the functional pathways (hazard ratio, 1.78; 95% CI, 1.38–2.31; FDR = 0.02; two-tailed Wald test for Cox regression coefficient) in the age-adjusted and sex-adjusted Cox models. However, this finding is not informative, because that classification is merely the total abundance of genes that did not contribute to any pathways for *E. hallii*[23]. No pathways remained significant after adjusting for multiple covariates independent of whether the pathway activities were analysed using dichotomized or inverse-ranked normalized data (Supplemental Tables S9 and S10).

## DISCUSSION

This study is the first to investigate the association of the gut microbiome with incident hypertension in a large, prospective, longitudinal cohort. Over a median follow-up of 19.8 years, 20% of the 3311 study participants developed hypertension. The overall gut microbiome composition of participants who had developed hypertension 20 years later was similar to people who did not develop hypertension. We also did not observe microbial taxa or functional profiles associated with risk of hypertension later in life.

Large cohort studies such as Coronary Artery Risk Development in Young Adults, CARDIA (*n* = 529), Estonian Microbiome Cohort (*n* = 2509), TwinsUK (*n* = 2737), Healthy Life In an Urban Setting (HELIUS, *n* = 4672), and FINRISK 2002 (*n* = 6953) have previously examined the cross-sectional associations between the gut microbiome and blood pressure [34–38]. The Estonian Microbiome Cohort reported gut dysbiosis to be significantly associated with hypertension. However, the machine learning prediction model whereby microbiome is included as a predictor for disease did not perform any better than conventional predictors such as age, sex, and BMI [35]. In the HELIUS study, the authors also used machine learning model to explain variance of gut microbiome and blood pressure. This approach explained 4% of blood pressure variance in age-adjusted and sex-adjusted models but only 2.1–2.2% after adjusting for covariates [37].

The CARDIA study reported positive associations between the gut microbiome and hypertension, but the significance of this association attenuated greatly after adjusting for covariates, and especially for BMI [34]. We observed a similarly strong association of BMI to the gut microbiome in our sensitivity analysis. Obesity is corollary to hypertension, both strong risk factors for metabolic syndrome and can eventually lead to cardiovascular diseases. Studies have shown obesity to be collectively associated with hypertension and with the gut microbiome [7,14]. Gut microbiome dysbiosis in association with obesity reflects metabolic dysfunction. BMI should thus always be included as a covariate to disentangle obesity from the outcome of interest, in this case, hypertension. For example, the TwinsUK study reported associations between gut microbiome and common diseases, but after adjusting for BMI, gut microbiome was not related to prevalent hypertension [39]. In a cross-sectional study of gut microbiome and blood pressure using FINRISK 2002 data, the number of genera associated with blood pressure was reduced by 59% after adjusting for BMI. These cross-sectional studies seem to imply that although a cross-sectional association between blood pressure and gut microbes exists, the inclusion of BMI as a covariate strongly attenuates this association. It also appears that gut microbiome features in obesity may have a strong association with dietary fibres, thus the importance of adjusting for dietary effects in gut microbiome models as we have done in this study [7,40,41].

Animal studies have also demonstrated that gut microbes can modulate blood pressure [10,12,42]. In a study by Li *et al.* [43] in 2017, germ-free mice developed elevated blood pressure after receiving faecal microbiota transplant from a hypertensive human donor, clearly demonstrating that gut microbes are involved in mediating blood pressure changes. Furthermore, a recent study demonstrated that administration of two probiotic strains effectively replenished bacterial strains lost in hypertensive mice, thereby mitigating fructose-induced hypertension [44]. Evidence is also pointing towards microbe-mediated metabolites being drivers of host blood pressure through the renin–angiotensin–aldosterone-related and immune-related pathways [12,45]. However, despite these promising results from in-vivo and in-vitro studies suggesting a role for microbial mechanistic pathways and microbe-mediated metabolites in modulating host blood pressure, human studies have not always agreed [42]. A preliminary interventional trial involving 20 treatment-naive hypertension patients demonstrated that consuming prebiotic starch released high levels of acetate and butyrate and observed a decrease in 24-h blood pressure [45]. Conversely, oral administration of butyrate increased 24-h blood pressure in a double-blind randomized placebo-controlled trial that involved 23 hypertension patients [46]. Our prospective cohort study did not find strong evidence of gut microbial taxa or functional pathways associated with incident hypertension. However, this does not mean that such taxa are not associated with current hypertension, either as cause or effect, and may be a point for future research.

Human microbiome sequencing and analysis techniques are rapidly improving. Therefore, despite conflicting prior reports and the negative results from this study, further research in this area is still needed. Future studies should focus on improving the robustness of study designs for a more efficient translation from association studies and animal models to human intervention studies. For example, animal studies could be designed to more accurately reflect actual human physiology, overcoming earlier design flaws [47]. Multilaboratory and international collaborations should also be encouraged to reduce the number of underpowered clinical human studies [47].

Common limitations of microbiome-health association studies, such as a small sample size, the use of short read sequencing, and 16S amplicon sequencing instead of metagenome sequencing, have increasingly been addressed over the years, and this current study has the novel aspect of being the first prospective cohort study in this domain [34,37–39]. However, we also acknowledge that the study sample was drawn from the Finnish general population, thus limiting the global generalizability of our results. Nonetheless, the overall composition is still fairly similar to gut microbiome compositions from Western countries where the top genera identified were *Bacteroides, Prevotella*, and *Alistipes*[19,48,49]. Another limitation of the study is that information about stool consistency, a known confounder in microbiome research was not collected in 2002 during the baseline examination [50].

Also, the microbiome data were shallow sequenced with short-read technology, making functional annotation challenging and potentially resulting in some pathways being underrepresented or even missed. In addition, we did not have gut or plasma metabolite data available for more detailed functional characterization. Stool sampling and sequencing were also only performed at a single time point in the year 2002. Several studies have shown that the gut microbiome is highly stable over the long term, especially in the absence of fatty liver disease and diabetes [51,52]. However, repeated stool sampling could improve the study design and increase the odds of detecting associations between the gut microbiome and incident cardiovascular disease should such associations exist [53]. The study design is also limited with the absence of absolute abundances of microbial taxa, which could provide more insights. Finally, clinical re-examinations were not performed in the present project. For a more accurate assessment of incident hypertension, repeated measurement of blood pressure would be preferred to confirm the diagnosis. Instead, we relied on national hospital discharge and drug reimbursement registers for diagnoses of incident hypertension, which may lead to under diagnosis of incident hypertension cases.

In conclusion, in this first-ever prospective study with long-term follow-up on the associations between the gut microbiome and incident hypertension, we observed a weak association in age-adjusted and sex-adjusted models but no associations in multivariable-adjusted models. We observed eight microbial genera associated with incident hypertension in age-adjusted and sex-adjusted Cox models. *CAG-628* was associated with incident hypertension when an age-adjusted and sex-adjusted ANCOM-BC2 model was used. Functional pathways were also not associated with incident hypertension. Our prospective cohort study does not support a strong association between the gut microbiome and risk of future hypertension.

## ACKNOWLEDGEMENTS

We thank all participants of the FINRISK 2002 survey for their contributions to this work, and Tara Schwartz for assistance with laboratory work, and Kari Koponen for assistance with the Healthy Food Choices index.

Data availability statement: the FINRISK data for the present study are available with a written application to the THL Biobank as instructed on the website of the Biobank (https://thl.fi/en/research-and-development/thl-biobank/for-researchers). A separate permission is needed from FINDATA (https://www.findata.fi/en/) for use of the electronic health record data. Metagenomic data are available through the European Genome–Phenome Archive (EGAD00001007035).

Author's contributions: T.N., R.K., and L.L. contributed to the conception and/or design of the study. L.F.Y. and J.P. contributed to analysis, interpretation, and visualization of the work. A.H., K.P., and V.S. contributed to acquisition and processing of data. L.F.Y. and T.N. drafted the manuscript. All authors read, critically revised the manuscript, and gave final approval for the manuscript. L.F.Y. and T.N. take final responsibility for the submission of the study for publication.

Funding: L.F.Y. has received a research grant co-funded by the European Union's Horizon Europe Framework programme for research and innovation 2021–2027 under the Marie Skłodowska-Curie grant agreement No 101126611. J.P. has received research grants from the Paavo Nurmi Foundation and the Finnish Medical Foundation. K.P. has received a postdoctoral grant [348439, 368511] from the Research Council of Finland. V.S. has received a research grant from the Juho Vainio Foundation. T.N. has received funding for this work from the Finnish Research Council, the Sigrid Jusélius Foundation, the Finnish Foundation for Cardiovascular Research and the Wellbeing Services County of Southwest Finland.

### Conflicts of interest

R.K. is a scientific advisory board member, and consultant for BiomeSense, Inc., has equity and receives income. He is a scientific advisory board member and has equity in GenCirq. He is a consultant for DayTwo, and receives income. He has equity in and acts as a consultant for Cybele. He is a co-founder of Biota, Inc., and has equity. He is a cofounder of Micronoma, and has equity and is a scientific advisory board member. T.N. has received consulting and authoring fees from AstraZeneca and Orion Corporation. 

## Supplementary Material

Supplemental Digital Content

## References

[1] WHO. Cardiovascular diseases Fact Sheet. Available at: https://www.who.int/news-room/fact-sheets/detail/cardiovascular-diseases-(cvds). 2021.

[2] Vaduganathan M Mensah GA Turco JV Fuster V Roth GA. The global burden of cardiovascular diseases and risk. *J Am Coll Cardiol* 2022; 80:2361–2371. 36368511 10.1016/j.jacc.2022.11.005

[3] Working group appointed by the Finnish Medical Association Duodecim, Finnish Blood Pressure Association. Elevated blood pressure. Current treatment recommendation. Available at:www.kaypahoito.fi. 2020.

[4] Laatikainen T, Niiranen T, Lehtoranta L, Jousilahti P. Verenpaine. Available at: https://www.thl.fi/tervesuomi_verkkoraportit/ilmioraportit_2023/verenpaine.html. 2023. https://www.thl.fi/tervesuomi_verkkoraportit/ilmioraportit_2023/verenpaine.html. (Accessed 3 September 2025)

[5] Gelfand EV Cannon CP. Antibiotics for secondary prevention of coronary artery disease: an ACES hypothesis but we need to PROVE IT. *Am Heart J* 2004; 147:202–209. 14760314 10.1016/j.ahj.2003.09.011

[6] Fromentin S Forslund SK Chechi K Aron-Wisnewsky J Chakaroun R Nielsen T. Microbiome and metabolome features of the cardiometabolic disease spectrum. *Nat Med* 2022; 28:303–314. 35177860 10.1038/s41591-022-01688-4 PMC8863577

[7] Chakaroun RM Olsson LM Bäckhed F. The potential of tailoring the gut microbiome to prevent and treat cardiometabolic disease. *Nat Rev Cardiol* 2023; 20:217–235. 36241728 10.1038/s41569-022-00771-0

[8] Hellman T Yeo L-F Palmu J Havulinna A Jousilahti P Laitinen V. Gut microbiome as a risk factor for future CKD. *Kidney Int Rep* 2025; 10:1673–1682. 40630297 10.1016/j.ekir.2025.04.007 PMC12231020

[9] Yano Y Niiranen TJ. Gut microbiome over a lifetime and the association with hypertension. *Curr Hypertens Rep* 2021; 23:15. 33686539 10.1007/s11906-021-01133-w

[10] Palmu J Lahti L Niiranen T. Targeting gut microbiota to treat hypertension: a systematic review. *Int J Environ Res Public Health* 2021; 18:1248. 33561095 10.3390/ijerph18031248 PMC7908114

[11] Mishima E Abe T. Role of the microbiota in hypertension and antihypertensive drug metabolism. *Hypertens Res* 2022; 45:246–253. 34887530 10.1038/s41440-021-00804-0

[12] Avery EG Bartolomaeus H Maifeld A Marko L Wiig H Wilck N. The gut microbiome in hypertension. *Circ Res* 2021; 128:934–950. 33793332 10.1161/CIRCRESAHA.121.318065

[13] Cai M Lin L Jiang F Peng Y Li S Chen L. Gut microbiota changes in patients with hypertension: a systematic review and meta-analysis. *J Clin Hypertens* 2023; 25:1053–1068. 10.1111/jch.14722 PMC10710550 37853925

[14] Trøseid M Andersen GØ Broch K Hov JR. The gut microbiome in coronary artery disease and heart failure: current knowledge and future directions. *EBioMedicine* 2020; 52:102649. 32062353 10.1016/j.ebiom.2020.102649 PMC7016372

[15] Althubaiti A. Information bias in health research: definition, pitfalls, and adjustment methods. *J Multidiscip Healthc* 2016; 9:211. 27217764 10.2147/JMDH.S104807 PMC4862344

[16] Joos R Boucher K Lavelle A Arumugam M Blaser MJ Claesson MJ. Examining the healthy human microbiome concept. *Nat Rev Microbiol* 2025; 23:192–205. 39443812 10.1038/s41579-024-01107-0

[17] Wang X Kattan MW. Cohort studies. *Chest* 2020; 158:S72–S78. 32658655 10.1016/j.chest.2020.03.014

[18] Borodulin K Tolonen H Jousilahti P Jula A Juolevi A Koskinen S. Cohort profile: the National FINRISK Study. *Int J Epidemiol* 2018; 47:696–1696. 29165699 10.1093/ije/dyx239

[19] Salosensaari A Laitinen V Havulinna AS Meric G Cheng S Perola M. Taxonomic signatures of cause-specific mortality risk in human gut microbiome. *Nat Commun* 2021; 12:2671. 33976176 10.1038/s41467-021-22962-y PMC8113604

[20] Earth Microbiome Project (EMP) high throughput (HTP) DNA extraction protocol. Available at: https://www.protocols.io/view/earth-microbiome-project-emp-high-throughput-htp-d-8epv5qqjv1bz/v1. (Accessed 26 August 2024)

[21] McDonald D Jiang Y Balaban M Cantrell K Zhu Q Gonzalez A. Greengenes2 unifies microbial data in a single reference tree. *Nat Biotechnol* 2023; 42:715–718. 37500913 10.1038/s41587-023-01845-1 PMC10818020

[22] Caspi R Billington R Keseler IM Kothari A Krummenacker M Midford PE. The MetaCyc database of metabolic pathways and enzymes - a 2019 update. *Nucleic Acids Res* 2020; 48:D445–D453. 31586394 10.1093/nar/gkz862 PMC6943030

[23] Beghini F McIver LJ Blanco-Míguez A Dubois L Asnicar F Maharjan S. Integrating taxonomic, functional, and strain-level profiling of diverse microbial communities with bioBakery 3. *Elife* 2021; 10:e65088. 33944776 10.7554/eLife.65088 PMC8096432

[24] Huang R Soneson C Ernst FGM Rue-Albrecht KC Yu G Hicks SC Robinson MD. TreeSummarizedExperiment: a S4 class for data with hierarchical structure. *F1000Res* 2021; 9:1246. 10.12688/f1000research.26669.1 PMC7684683 33274053

[25] Ernst F, Shetty S, Borman T, Lahti L. microbiome/mia: Microbiome analysis. 2024. Available at: https://github.com/microbiome/mia. (Accessed 20 September 2024.

[26] Lahti L, Borman T, Ernst FG. Orchestrating Microbiome Analysis. 2024. Available at: https://microbiome.github.io/OMA/docs/devel/. (Accessed 20 September 2024)

[27] Sund R. Quality of the Finnish Hospital Discharge Register: a systematic review. *Scand J Public Health* 2012; 40:505–515. 22899561 10.1177/1403494812456637

[28] Koponen KK Salosensaari A Ruuskanen MO Havulinna AS Männistö S Jousilahti P. Associations of healthy food choices with gut microbiota profiles. *Am J Clin Nutr* 2021; 114:605–616. 34020448 10.1093/ajcn/nqab077 PMC8326043

[29] Vuori MA Laukkanen JA Pietilä A Havulinna AS Kähönen M Salomaa V Niiranen TJ. FinnGen investigators. The validity of heart failure diagnoses in the Finnish Hospital Discharge Register. *Scand J Public Health* 2020; 48:20–28. 31068116 10.1177/1403494819847051

[30] Yuan L Li Y Chen M Xue L Wang J Ding Y. Therapeutic applications of gut microbes in cardiometabolic diseases: current state and perspectives. *Appl Microbiol Biotechnol* 2024; 108:156. 38244075 10.1007/s00253-024-13007-7 PMC10799778

[31] Cao Q Sun X Rajesh K Chalasani N Gelow K Katz B. Effects of rare microbiome taxa filtering on statistical analysis. *Front Microbiol* 2021; 11:607325. 33510727 10.3389/fmicb.2020.607325 PMC7835481

[32] Anderson MJ. Permutational multivariate analysis of variance (PERMANOVA). *Wiley StatsRef: Statistics Reference Online* 2017; Wiley, 1–15.

[33] Lin H Peddada SD. Multigroup analysis of compositions of microbiomes with covariate adjustments and repeated measures. *Nat Methods* 2024; 21:83–91. 38158428 10.1038/s41592-023-02092-7 PMC10776411

[34] Sun S Lulla A Sioda M Winglee K Wu MC Jacobs DR. Gut microbiota composition and blood pressure: Coronary Artery Risk Development in Young Adults (CARDIA) Study. *Hypertension* 2019; 73:998. 30905192 10.1161/HYPERTENSIONAHA.118.12109 PMC6458072

[35] Aasmets O Krigul KL Lüll K Metspalu A Org E. Gut metagenome associations with extensive digital health data in a volunteer-based Estonian microbiome cohort. *Nat Commun* 2022; 13:869. 35169130 10.1038/s41467-022-28464-9 PMC8847343

[36] Louca P Mompeo O Leeming ER Berry SE Mangino M Spector TD. Dietary influence on systolic and diastolic blood pressure in the TwinsUK Cohort. *Nutrients* 2020; 12:1–12. 10.3390/nu12072130 PMC7400881 32708992

[37] Verhaar BJH Collard D Prodan A Levels JHM Zwinderman AH Backhed F. Associations between gut microbiota, faecal short-chain fatty acids, and blood pressure across ethnic groups: the HELIUS study. *Eur Heart J* 2020; 41:4259–4267. 32869053 10.1093/eurheartj/ehaa704 PMC7724641

[38] Palmu J Salosensaari A Havulinna AS Cheng S Inouye M Jain M. Association between the gut microbiota and blood pressure in a population cohort of 6953 individuals. *J Am Heart Assoc* 2020; 9:e016641. 32691653 10.1161/JAHA.120.016641 PMC7792269

[39] Jackson MA Verdi S Maxan ME Shin CM Zierer J Bowyer RCE. Gut microbiota associations with common diseases and prescription medications in a population-based cohort. *Nat Commun* 2018; 9:1–8. 29985401 10.1038/s41467-018-05184-7 PMC6037668

[40] Delzenne NM Bindels LB Neyrinck AM Walter J. The gut microbiome and dietary fibres: implications in obesity, cardiometabolic diseases and cancer. *Nat Rev Microbiol* 2025; 23:225–238. 39390291 10.1038/s41579-024-01108-z

[41] Duncan SH Belenguer A Holtrop G Johnstone AM Flint HJ Lobley GE. Reduced dietary intake of carbohydrates by obese subjects results in decreased concentrations of butyrate and butyrate-producing bacteria in feces. *Appl Environ Microbiol* 2007; 73:1073–1078. 17189447 10.1128/AEM.02340-06 PMC1828662

[42] O’Donnell JA Zheng T Meric G Marques FZ. The gut microbiome and hypertension. *Nat Rev Nephrol* 2023; 19:153–167. 36631562 10.1038/s41581-022-00654-0

[43] Li J Zhao F Wang Y Chen J Tao J Tian G. Gut microbiota dysbiosis contributes to the development of hypertension. *Microbiome* 2017; 5:14. 28143587 10.1186/s40168-016-0222-x PMC5286796

[44] Zhang Y Zheng T Ma D Shi P Zhang H Li J. Probiotics Bifidobacterium lactis M8 and Lactobacillus rhamnosus M9 prevent high blood pressure via modulating the gut microbiota composition and host metabolic products. *mSystems* 2023; 8:e0033123. 37855616 10.1128/msystems.00331-23 PMC10734487

[45] Jama HA Rhys-Jones D Nakai M Yao CK Climie RE Sata Y. Prebiotic intervention with HAMSAB in untreated essential hypertensive patients assessed in a phase II randomized trial. *Nat Cardiovasc Res* 2023; 2:35–43. 39196205 10.1038/s44161-022-00197-4

[46] Verhaar BJH Wijdeveld M Wortelboer K Rampanelli E Levels JHM Collard D. Effects of oral butyrate on blood pressure in patients with hypertension: a randomized, placebo-controlled trial. *Hypertension* 2024; 81:2124–2136. 39034917 10.1161/HYPERTENSIONAHA.123.22437 PMC11404767

[47] Ineichen BV Furrer E Grüninger SL Zürrer WE Macleod MR. Analysis of animal-to-human translation shows that only 5% of animal-tested therapeutic interventions obtain regulatory approval for human applications. *PLoS Biol* 2024; 22:e3002667. 38870090 10.1371/journal.pbio.3002667 PMC11175415

[48] Arumugam M Raes J Pelletier E Le Paslier D Yamada T Mende DR. Enterotypes of the human gut microbiome. *Nature* 2011; 473:174–180. 21508958 10.1038/nature09944 PMC3728647

[49] Human Microbiome Project Consortium. Structure, function and diversity of the healthy human microbiome. *Nature* 2012; 486:207–214. 22699609 10.1038/nature11234 PMC3564958

[50] Vandeputte D Falony G Vieira-Silva S Tito RY Joossens M Raes J. Stool consistency is strongly associated with gut microbiota richness and composition, enterotypes and bacterial growth rates. *Gut* 2016; 65:57–62. 26069274 10.1136/gutjnl-2015-309618 PMC4717365

[51] Frost F Kacprowski T Rühlemann M Pietzner M Bang C Franke A. Long-term instability of the intestinal microbiome is associated with metabolic liver disease, low microbiota diversity, diabetes mellitus and impaired exocrine pancreatic function. *Gut* 2021; 70:522–530. 33168600 10.1136/gutjnl-2020-322753 PMC7873430

[52] Zhou X Shen X Johnson JS Spakowicz DJ Agnello M Zhou W. Longitudinal profiling of the microbiome at four body sites reveals core stability and individualized dynamics during health and disease. *Cell Host Microbe* 2024; 32:506.e9–526.e9. 38479397 10.1016/j.chom.2024.02.012 PMC11022754

[53] Vandeputte D De Commer L Tito RY Kathagen G Sabino J Vermeire S. Temporal variability in quantitative human gut microbiome profiles and implications for clinical research. *Nat Commun* 2021; 12:6740. 34795283 10.1038/s41467-021-27098-7 PMC8602282

