# Supplementary material for: Prospective association between the gut microbiome and incident hypertension: a 20-year cohort study
Source: J Hypertens. 2026 Feb 9;44(4):673–81. doi: 10.1097/HJH.0000000000004254 (PMC12955955; doi:10.1097/HJH.0000000000004254)
Supplement: Supplemental Digital Content [file jhype-44-673-s001.docx]

**Supplemental methods**

Sensitivity and power analysis

With 3311 participants who had 675 cases of incident hypertension and a two-sided alpha level of 0.05, the study is 80% powered to detect hazard ratios of 1.293 and 1.011 for 1-unit increases in the Shannon index (standard deviation 0.42) and systolic blood pressure (standard deviation 9.9), respectively.[30] A sensitivity analysis was performed to check for selection bias between participants who were included against those excluded for incomplete covariates. Differences in covariates between participants with and without incident hypertension were tested using Chi-square test for categorical variables, and Student’s T-test for continuous variables. A sensitivity analysis was performed to examine the inclusion of systolic blood pressure into the Cox model for both alpha diversity and differential abundance analyses. Furthermore, a sensitivity analysis was performed on the multivariable-adjusted associations of alpha diversity and species-level relative abundances with incident hypertension using Cox models, while limiting the follow-up time to 10 years.

**Supplemental results**

Sensitivity analysis

Participants who developed hypertension had significantly higher baseline blood pressure (p < 0.001, **Table 1**) thus as a sensitivity analysis, we assessed the inclusion of systolic blood pressure into the Cox model. Alpha diversity of participants with incident hypertension remained insignificant with the inclusion of systolic blood pressure as an additional covariate in multivariable-adjusted Cox model (HR, 1.00; 95% CI, 0.93–1.08; p=0.91).

We also assessed the additional of systolic blood pressure into the Cox model for differential abundance analysis and found no taxa to be significantly different in association with incident hypertension (FDR > 0.05).

As BMI was shown to be a strong confounder in gut microbiome studies, we tested this by removing BMI from the multivariable-adjusted model. For alpha diversity, we observed that although the association between alpha diversity (Shannon index) and incident hypertension remained non-significant, the P value decreased from 0.84 to 0.12 (**Supplemental Figure S11**). This suggests that BMI is a strong confounder and should be included as a covariate.

We also performed a sensitivity analysis while restricting the follow-up time to 10 years. A total of 226 participants developed hypertension during this time. With a shorter follow-up time, we observed no significant differences of alpha diversity (Shannon index) or individual taxa with abundances incident hypertension There was also no significant species found in multivariable adjusted Cox models (FDR > 0.05 for all, **Supplemental table S12**).

**Supplemental Table S1.** Definitions of register-based comorbidities and outcomes.

| Comorbidity/Outcome | Hospital discharge and death registers | Drug reimbursement and purchase registers |
| --- | --- | --- |
| Diabetes | ICD-10: E10-E14 ICD-9: 250 ICD-8: 250 | ATC: A10, A10A, A10B  DR: 103 |
| Cardiovascular disease (*As a primary or secondary diagnosis code) | Hospital discharge register:  CD-10: I200, I21*, I22*, I61, I63* (not I63.6), I64*  ICD-9: 410*, 4110, 431*, 4330A*, 4331A*, 4339A*, 4340A*, 4341A*, 4349A*, 436*  ICD-8: 410* 4110, 431* (not 43101 or 43191) 433*, 434*, 436*  Procedure code for PCI or CABG  Causes of death register:  ICD-10: I20-I25, I21-I22* I46, R96, R98, I61*, I63* (not I63.6), I64*  ICD-9: 410-414, 410*, 798 (not 7980A), 431*, 4330A*, 4331A*, 4339A*, 4340A*, 4341A*, 4349A*, 436*  ICD-8: 410-414, 798 (not 7980A), 431* (not 43101 or 43191) 433*, 434*, 436* |  |
| Hypertension | ICD-10: I10-I15, I67.4 ICD-9: 4019X, 4029A, 4029B, 4039A, 4040A, 4059A, 4059B, 4372A, 4059X ICD-8: 400, 401, 402, 403, 404 | ATC: C03A, C03BA11, C03EA01, C07B, C09BA, C09DA, C07FB, C08CA, C08GA, C09BB, C09BX01, C09BX03, C09BX04, C09DB, C09DX, C09XA  DR: 205 |

ICD, International Classification of Diseases; ATC, Anatomical therapeutic chemical code; CABG, coronary artery bypass grafting; DR, Drug reimbursement code; PCI, percutaneous coronary intervention.

**Supplemental table S2.** Sensitivity analysis to assess for selection bias by testing the differences in baseline characteristics among participants with complete and incomplete covariates.

|  | Included participants | Excluded participants | P |
| --- | --- | --- | --- |
| n | 3311 | 474 |  |
| Age (mean (SD)) | 43.42 (11.86) | 44.41 (12.53) | 0.09 |
| BMI (mean (SD)) | 25.59 (4.12) | 25.95 (3.86) | 0.07 |
| Systolic BP (mean (SD)) | 122.12 (9.88) | 122.16 (10.15) | 0.94 |
| Diastolic BP (mean (SD)) | 73.63 (8.55) | 74.51 (8.18) | 0.04 |
| Healthy food choices (mean (SD)) | 190.98 (86.40) | 172.02 (93.72) | 0.41 |
| Shannon (mean (SD)) | 4.12 (0.42) | 4.08 (0.42) | 0.02 |
| Observed (mean (SD)) | 1060.40 (211.16) | 1042.34 (235.21) | 0.09 |

**Supplemental Table S3.** Association between beta diversity and each covariate (PERMANOVA; Bray-Curtis index at species level).

|  | DF | Sums of square | F | P | Total variance | Total variance |
| --- | --- | --- | --- | --- | --- | --- |
| Model | 8 | 10.79 | 7.35 | 0.001 | 617.07 | 0.017 |
| Age | 1 | 2.15 | 11.70 | 0.001 | 617.07 | 0.003 |
| Sex | 1 | 2.44 | 13.29 | 0.001 | 617.07 | 0.004 |
| BMI | 1 | 1.92 | 10.46 | 0.001 | 617.07 | 0.003 |
| Diabetes | 1 | 0.29 | 1.58 | 0.031 | 617.07 | <0.001 |
| Healthy food choices | 1 | 0.75 | 4.06 | 0.001 | 617.07 | 0.001 |
| Cardiovascular disease | 1 | 0.21 | 1.13 | 0.255 | 617.07 | <0.001 |
| Smoking | 1 | 1.44 | 7.87 | 0.001 | 617.07 | 0.002 |
| Incident hypertension | 1 | 0.17 | 0.92 | 0.559 | 617.07 | <0.001 |
| Residual | 3302 | 606.28 | NA | NA | 617.07 | 0.983 |

DF, degree of freedom; F, F-statistic; BMI, Body mass index

**Supplemental Table S4.** Associations between the relative abundance of bacterial families (the top 10 based on FDR) and incident hypertension in Cox proportional hazards models.

| Family | HR | 95% CI | FDR |
| --- | --- | --- | --- |
| Age- and sex-adjusted model | | | |
| Alkalibacillaceae | 1.08 | 1.02 — 1.13 | 0.04 |
| Anaerovoracaceae | 1.22 | 1.06 — 1.39 | 0.04 |
| Butyricicoccaceae | 1.18 | 1.06 — 1.32 | 0.04 |
| CAG_138 | 0.89 | 0.83 — 0.96 | 0.04 |
| Eggerthellaceae | 1.12 | 1.04 — 1.21 | 0.04 |
| UBA660 | 0.94 | 0.91 — 0.98 | 0.04 |
| Acidaminococcaceae | 1.05 | 1.01 — 1.10 | 0.09 |
| Erysipelotrichaceae | 1.14 | 1.02 — 1.27 | 0.11 |
| Coriobacteriaceae | 1.06 | 1.01 — 1.11 | 0.11 |
| CAG_508 | 0.94 | 0.89 — 0.99 | 0.13 |
| Multivariable-adjusted model | | | |
| Alkalibacillaceae | 1.06 | 1.01 — 1.11 | 0.63 |
| CAG_312 | 1.05 | 1.01 — 1.09 | 0.63 |
| Acidaminococcaceae | 1.02 | 0.98 — 1.07 | 0.93 |
| Acutalibacteraceae | 0.94 | 0.86 — 1.03 | 0.93 |
| Akkermansiaceae | 1.00 | 0.96 — 1.04 | 0.93 |
| Anaerotignaceae | 1.02 | 0.94 — 1.11 | 0.93 |
| Anaerovoracaceae | 1.08 | 0.95 — 1.24 | 0.93 |
| Bacteroidaceae | 0.99 | 0.92 — 1.07 | 0.93 |
| Barnesiellaceae | 0.98 | 0.92 — 1.04 | 0.93 |
| Bifidobacteriaceae | 0.98 | 0.93 — 1.03 | 0.93 |

HR, hazard ratio; CI, confidence interval; FDR, false discovery rate with Benjamini-Hochberg corrected P value. Multivariable-adjusted models were adjusted for age, sex, BMI, diabetes, healthy food choices, cardiovascular disease and smoking.

**Supplemental Table S5.** Associations between the relative abundance of bacterial species (the top 10 based on FDR) and incident hypertension in Cox proportional hazards models.

| Species | HR | 95% CI | FDR |
| --- | --- | --- | --- |
| Age- and sex-adjusted model | | | |
| *Blautia_A_141780_hansenii* | 1.21 | 1.10 — 1.33 | 0.02 |
| *Lachnospira eligens* | 0.90 | 0.85 — 0.95 | 0.04 |
| *Blautia_A_141781_wexlerae* | 1.14 | 1.06 — 1.23 | 0.04 |
| *CAG_317_sp000433215* | 1.25 | 1.10 — 1.42 | 0.04 |
| *CAG_628_sp003524085* | 0.94 | 0.91 — 0.98 | 0.04 |
| *Mediterraneibacter_A_155590_butyricigenes* | 1.25 | 1.10 — 1.43 | 0.04 |
| *Oliverpabstia intestinalis* | 1.15 | 1.05 — 1.26 | 0.06 |
| *Prevotella sp003447235* | 1.05 | 1.02 — 1.08 | 0.06 |
| *Ruminococcus_B_gnavus* | 1.08 | 1.03 — 1.14 | 0.06 |
| *Anaerostipes hadrus* | 1.10 | 1.03 — 1.18 | 0.07 |
| Multivariable-adjusted model | | | |
| *Eubacterium_G_ventriosum* | 0.88 | 0.82 — 0.94 | 0.07 |
| *Merdousia gallistercoris* | 1.05 | 1.02 — 1.09 | 0.47 |
| *Allobacillus sp007559425* | 1.07 | 1.01 — 1.13 | 0.72 |
| *Anaerostipes hadrus* | 1.08 | 1.01 — 1.15 | 0.72 |
| *Bifidobacterium adolescentis* | 0.96 | 0.93 — 0.99 | 0.72 |
| *Lachnospira eligens* | 0.93 | 0.88 — 0.99 | 0.72 |
| *Prevotella sp003447235* | 1.04 | 1.01 — 1.07 | 0.72 |
| *Ruminococcus_C_58660_callidus* | 0.92 | 0.86 — 0.99 | 0.72 |
| *Senegalimassilia faecalis* | 1.05 | 1.01 — 1.09 | 0.72 |
| *Ellagibacter isourolithinifaciens* | 1.03 | 1.00 — 1.06 | 0.80 |

HR, hazard ratio; CI, confidence interval; FDR, false discovery rate with Benjamini-Hochberg corrected P value. Multivariable-adjusted models were adjusted for age, sex, BMI, diabetes, healthy food choices, cardiovascular disease and smoking.

**Supplemental Table S6.** Associations between the relative abundance of bacterial genera (the top 10 based on FDR) and incident hypertension in ANCOM-BC2.

| Genus | LFC | SE | FDR | Sensitivity test* |
| --- | --- | --- | --- | --- |
| Age- and sex-adjusted model | | | | |
| *Scatocola* | -0.41 | 0.11 | 0.02 | FALSE |
| *CAG-628* | -0.34 | 0.10 | 0.04 | TRUE |
| *Faecenecus* | -0.28 | 0.08 | 0.05 | FALSE |
| *Methanobrevibacter_A* | -0.29 | 0.09 | 0.06 | FALSE |
| *Intestinimonas* | -0.11 | 0.04 | 0.26 | TRUE |
| *Fimivivens* | -0.13 | 0.05 | 0.29 | TRUE |
| *PeH17* | -0.16 | 0.07 | 0.29 | TRUE |
| *Ruminococcus_B* | 0.17 | 0.07 | 0.29 | TRUE |
| *Senegalimassilia* | 0.22 | 0.09 | 0.29 | TRUE |
| *CAG-273* | -0.22 | 0.09 | 0.29 | FALSE |
| Multivariable-adjusted model | | | | |
| *Scatocola* | -0.34 | 0.11 | 0.35 | FALSE |
| *CAG-345* | 0.27 | 0.10 | 0.44 | FALSE |
| *Anaerostipes* | 0.13 | 0.06 | 0.79 | TRUE |
| *CAG-288* | 0.19 | 0.09 | 0.79 | FALSE |
| *Ellagibacter* | 0.26 | 0.11 | 0.79 | FALSE |
| *Merdousia* | 0.22 | 0.10 | 0.79 | TRUE |
| *Senegalimassilia* | 0.19 | 0.09 | 0.79 | TRUE |
| *14-Feb* | 0.01 | 0.04 | 0.95 | TRUE |
| *51-20* | -0.02 | 0.09 | 0.95 | TRUE |
| *Acetatifactor* | -0.08 | 0.06 | 0.95 | TRUE |

LFC, natural log fold change, SE, standard error, FDR, false discovery rate with Benjamini-Hochberg corrected P value. Multivariable-adjusted models were adjusted for age, sex, BMI, diabetes, healthy food choices, cardiovascular disease and smoking.

*ANCOM-BC2 applies a sensitivity analysis for pseudo-count addition. If the test is false, that means the taxa failed the sensitivity test against pseudo-count addition. Thus, even if there was a significant FDR value, it is likely to be a false positive from the pseudo-count addition and not true difference in abundance.

**Supplemental Table S7.** Associations between the relative abundance of bacterial species (the top 10 based on FDR) and incident hypertension in ANCOM-BC2.

| Species | LFC | SE | FDR | Sensitivity test* |
| --- | --- | --- | --- | --- |
| Age- and sex-adjusted model | | | | |
| *Scatocola faecipullorum* | -0.39 | 0.11 | 0.06 | FALSE |
| *CAG-628 sp003524085* | -0.32 | 0.10 | 0.10 | TRUE |
| *Faecenecus gallistercoris* | -0.27 | 0.08 | 0.10 | FALSE |
| *Blautia_A_141781 wexlerae* | 0.14 | 0.05 | 0.36 | TRUE |
| *Lachnospira eligens* | -0.18 | 0.07 | 0.36 | TRUE |
| *Ruminococcus_B gnavus* | 0.19 | 0.07 | 0.36 | TRUE |
| *Senegalimassilia faecalis* | 0.23 | 0.09 | 0.36 | TRUE |
| *Anaerostipes hadrus* | 0.15 | 0.06 | 0.36 | TRUE |
| *Blautia_A_141780 hansenii* | 0.12 | 0.05 | 0.36 | TRUE |
| *Ellagibacter isourolithinifaciens* | 0.27 | 0.11 | 0.36 | TRUE |
| Multivariable-adjusted model | | | | |
| *Scatocola faecipullorum* | -0.33 | 0.11 | 0.62 | FALSE |
| *CAG-345 sp000433315* | 0.28 | 0.10 | 0.63 | FALSE |
| *Eubacterium_G ventriosum* | -0.16 | 0.06 | 0.68 | TRUE |
| *Merdousia gallistercoris* | 0.24 | 0.10 | 0.87 | TRUE |
| *Ellagibacter isourolithinifaciens* | 0.26 | 0.11 | 0.97 | FALSE |
| *51-20 sp001917175* | -0.05 | 0.09 | 0.99 | TRUE |
| *Acetatifactor sp900066565* | -0.08 | 0.06 | 0.99 | TRUE |
| *Adlercreutzia equolifaciens* | 0.00 | 0.08 | 0.99 | TRUE |
| *AF33-28 sp003477885* | 0.02 | 0.04 | 0.99 | TRUE |
| *Agathobacter faecis* | -0.02 | 0.07 | 0.99 | TRUE |

LFC, natural log fold change, SE, standard error, FDR, false discovery rate with Benjamini-Hochberg corrected P value. Multivariable-adjusted models were adjusted for age, sex, BMI, diabetes, healthy food choices, cardiovascular disease and smoking.

*ANCOM-BC2 applies a sensitivity analysis for pseudo-count addition. If the test is false, that means the taxa failed the sensitivity test against pseudo-count addition. Thus, even if there was a significant FDR value, it is likely to be a false positive from the pseudo-count addition and not true difference in abundance.

| Family | LFC | SE | FDR | Sensitivity test* |
| --- | --- | --- | --- | --- |
| Age- and sex-adjusted model | | | | |
| Methanobacteriaceae | -0.35 | 0.10 | 0.02 | FALSE |
| UBA660 | -0.27 | 0.11 | 0.19 | TRUE |
| CAG-138 | -0.18 | 0.07 | 0.19 | TRUE |
| Akkermansiaceae | -0.16 | 0.10 | 0.57 | TRUE |
| Eggerthellaceae | 0.09 | 0.06 | 0.57 | TRUE |
| Rikenellaceae | -0.12 | 0.07 | 0.57 | FALSE |
| Coriobacteriaceae | 0.16 | 0.09 | 0.57 | TRUE |
| Alkalibacillaceae | 0.17 | 0.09 | 0.57 | FALSE |
| Gastranaerophilaceae | -0.10 | 0.07 | 0.57 | TRUE |
| CAG-239 | -0.16 | 0.10 | 0.57 | TRUE |
| Multivariable-adjusted model | | | | |
| CAG-288 | 0.30 | 0.11 | 0.41 | FALSE |
| Acidaminococcaceae | 0.09 | 0.10 | 0.97 | TRUE |
| Acutalibacteraceae | -0.04 | 0.06 | 0.97 | TRUE |
| Akkermansiaceae | -0.01 | 0.10 | 0.97 | TRUE |
| Alkalibacillaceae | 0.12 | 0.08 | 0.97 | FALSE |
| Anaerotignaceae | 0.01 | 0.06 | 0.97 | TRUE |
| Anaerovoracaceae | 0.03 | 0.05 | 0.97 | TRUE |
| Bacteroidaceae | -0.01 | 0.06 | 0.97 | TRUE |
| Barnesiellaceae | -0.03 | 0.07 | 0.97 | TRUE |
| Bifidobacteriaceae | -0.01 | 0.08 | 0.97 | TRUE |

**Supplemental Table S8.** Associations between the relative abundance of bacterial families (the top 10 based on FDR) and incident hypertension in ANCOM-BC2.

LFC, natural log fold change, SE, standard error, FDR, false discovery rate with Benjamini-Hochberg corrected P value. Multivariable-adjusted models were adjusted for age, sex, BMI, diabetes, healthy food choices, cardiovascular disease and smoking.

*ANCOM-BC2 applies a sensitivity analysis for pseudo-count addition. If the test is false, that means the taxa failed the sensitivity test against pseudo-count addition. Thus, even if there was a significant FDR value, it is likely to be a false positive from the pseudo-count addition and not true difference in abundance.

**Supplemental Table S9.** Associations between the relative abundance of functional pathways (the top 10 based on FDR) and incident hypertension using dichotomized variables (presence/absence) in Cox model.

| Pathway | HR | 95% CI | FDR |
| --- | --- | --- | --- |
| Age- and sex-adjusted model |  |  |  |
| UNINTEGRATED\|g__Eubacterium.s__Eubacterium_hallii | 1.78 | 1.38 — 2.31 | 0.02 |
| UNINTEGRATED\|g__Dorea.s__Dorea_longicatena | 1.54 | 1.25 — 1.91 | 0.05 |
| PWY-2942: L-lysine biosynthesis III\|g__Blautia.s__Blautia_wexlerae | 1.73 | 1.30 — 2.28 | 0.06 |
| METH-ACETATE-PWY: methanogenesis from acetate\|g__Blautia.s__Blautia_wexlerae | 1.57 | 1.22 — 2.02 | 0.17 |
| ASPASN-PWY: superpathway of L-aspartate and L-asparagine biosynthesis\|g__Blautia.s__Blautia_obeum | 1.63 | 1.23 — 2.15 | 0.17 |
| PEPTIDOGLYCANSYN-PWY: peptidoglycan biosynthesis I (meso-diaminopimelate containing)\|g__Blautia.s__Blautia_wexlerae | 1.60 | 1.21 — 2.11 | 0.17 |
| PWY-6317: D-galactose degradation I (Leloir pathway)\|g__Blautia.s__Blautia_wexlerae | 1.60 | 1.21 — 2.11 | 0.17 |
| PWY66-422\|g__Blautia.s__Blautia_wexlerae | 1.60 | 1.21 — 2.11 | 0.17 |
| PWY-6387: UDP-N-acetylmuramoyl-pentapeptide biosynthesis I (meso-diaminopimelate containing)\|g__Blautia.s__Blautia_wexlerae | 1.55 | 1.19 — 2.02 | 0.17 |
| PWY-6703: preQ0 biosynthesis\|g__Blautia.s__Blautia_obeum | 1.59 | 1.20 — 2.11 | 0.17 |
| Multivariable-adjusted model |  |  |  |
| UNINTEGRATED\|g__Eubacterium.s__Eubacterium_hallii | 1.53 | 1.18 — 1.98 | 1 |
| UNINTEGRATED\|g__Lachnospira.s__Lachnospira_pectinoschiza | 0.71 | 0.56 — 0.90 | 1 |
| UNINTEGRATED\|g__Dorea.s__Dorea_longicatena | 1.35 | 1.09 — 1.67 | 1 |
| PWY-7221: guanosine ribonucleotides de novo biosynthesis\|g__Bacteroides.s__Bacteroides_stercoris | 0.68 | 0.51 — 0.90 | 1 |
| PWY-2942: L-lysine biosynthesis III\|g__Blautia.s__Blautia_wexlerae | 1.44 | 1.08 — 1.90 | 1 |
| ASPASN-PWY: superpathway of L-aspartate and L-asparagine biosynthesis\|g__Blautia.s__Blautia_obeum | 1.42 | 1.07 — 1.88 | 1 |
| PWY-7219: adenosine ribonucleotides de novo biosynthesis\|g__Dialister.s__Dialister_invisus | 1.30 | 1.05 — 1.61 | 1 |
| PWY0-1586: peptidoglycan maturation (meso-diaminopimelate containing)\|g__Bacteroides.s__Bacteroides_stercoris | 0.70 | 0.52 — 0.94 | 1 |
| PWY-7221: guanosine ribonucleotides de novo biosynthesis\|g__Fusicatenibacter.s__Fusicatenibacter_saccharivorans | 1.34 | 1.05 — 1.72 | 1 |
| PWY-6147: 6-hydroxymethyl-dihydropterin diphosphate biosynthesis I\|g__Bacteroides.s__Bacteroides_uniformis | 1.21 | 1.03 — 1.42 | 1 |

HR, hazard ratio, CI, confidence interval, FDR, false discovery rate with Benjamini-Hochberg corrected P value. Multivariable-adjusted models were adjusted for age, sex, BMI, diabetes, healthy food choices, cardiovascular disease and smoking.

**Supplemental Table S10.** Associations between the relative abundance of functional pathways (the top 10 based on FDR) and incident hypertension using inverse rank transformation in Cox model.

| Pathway | HR | 95% CI | FDR |
| --- | --- | --- | --- |
| Age- and sex-adjusted model | | | |
| UNINTEGRATED\|g__Eubacterium.s__Eubacterium_hallii | 1.29 | 1.14 — 1.46 | 0.06 |
| UNINTEGRATED\|g__Dorea.s__Dorea_longicatena | 1.24 | 1.12 — 1.39 | 0.06 |
| PWY-2942: L-lysine biosynthesis III\|g__Blautia.s__Blautia_wexlerae | 1.28 | 1.12 — 1.46 | 0.11 |
| METH-ACETATE-PWY: methanogenesis from acetate\|g__Blautia.s__Blautia_wexlerae | 1.25 | 1.10 — 1.41 | 0.14 |
| ASPASN-PWY: superpathway of L-aspartate and L-asparagine biosynthesis\|g__Blautia.s__Blautia_obeum | 1.25 | 1.10 — 1.42 | 0.14 |
| DTDPRHAMSYN-PWY: dTDP-&beta;-L-rhamnose biosynthesis\|g__Dorea.s__Dorea_longicatena | 1.24 | 1.09 — 1.40 | 0.14 |
| PWY-6121: 5-aminoimidazole ribonucleotide biosynthesis I\|g__Dialister.s__Dialister_invisus | 1.21 | 1.08 — 1.36 | 0.14 |
| PWY-7219: adenosine ribonucleotides de novo biosynthesis\|g__Dialister.s__Dialister_invisus | 1.20 | 1.08 — 1.35 | 0.14 |
| PWY-6527: stachyose degradation\|g__Blautia.s__Blautia_wexlerae | 1.22 | 1.08 — 1.38 | 0.14 |
| SER-GLYSYN-PWY: superpathway of L-serine and glycine biosynthesis I\|g__Dialister.s__Dialister_invisus | 1.22 | 1.08 — 1.38 | 0.14 |
| Multivariable-adjusted model | | | |
| UNINTEGRATED\|g__Eubacterium.s__Eubacterium_hallii | 1.20 | 1.06 — 1.36 | 1 |
| UNINTEGRATED\|g__Dorea.s__Dorea_longicatena | 1.16 | 1.04 — 1.29 | 1 |
| PWY-7221: guanosine ribonucleotides de novo biosynthesis\|g__Bacteroides.s__Bacteroides_stercoris | 0.82 | 0.71 — 0.96 | 1 |
| UNINTEGRATED\|g__Lachnospira.s__Lachnospira_pectinoschiza | 0.84 | 0.74 — 0.96 | 1 |
| PWY-7219: adenosine ribonucleotides de novo biosynthesis\|g__Dialister.s__Dialister_invisus | 1.15 | 1.03 — 1.28 | 1 |
| ASPASN-PWY: superpathway of L-aspartate and L-asparagine biosynthesis\|g__Blautia.s__Blautia_obeum | 1.17 | 1.03 — 1.33 | 1 |
| PWY-2942: L-lysine biosynthesis III\|g__Blautia.s__Blautia_wexlerae | 1.17 | 1.03 — 1.33 | 1 |
| PWY-6147: 6-hydroxymethyl-dihydropterin diphosphate biosynthesis I\|g__Bacteroides.s__Bacteroides_uniformis | 1.12 | 1.02 — 1.23 | 1 |
| PWY0-1586: peptidoglycan maturation (meso-diaminopimelate containing)\|g__Bacteroides.s__Bacteroides_stercoris | 0.84 | 0.72 — 0.97 | 1 |
| DTDPRHAMSYN-PWY: dTDP-&beta;-L-rhamnose biosynthesis\|g__Bacteroides.s__Bacteroides_stercoris | 0.85 | 0.74 — 0.98 | 1 |

HR, hazard ratio, CI, confidence interval, FDR, false discovery rate with Benjamini-Hochberg corrected P value. Multivariable-adjusted models were adjusted for age, sex, BMI, diabetes, healthy food choices, cardiovascular disease and smoking.

**Supplemental Figure S11.** Sensitivity analysis by excluding BMI from multivariable-adjusted model.


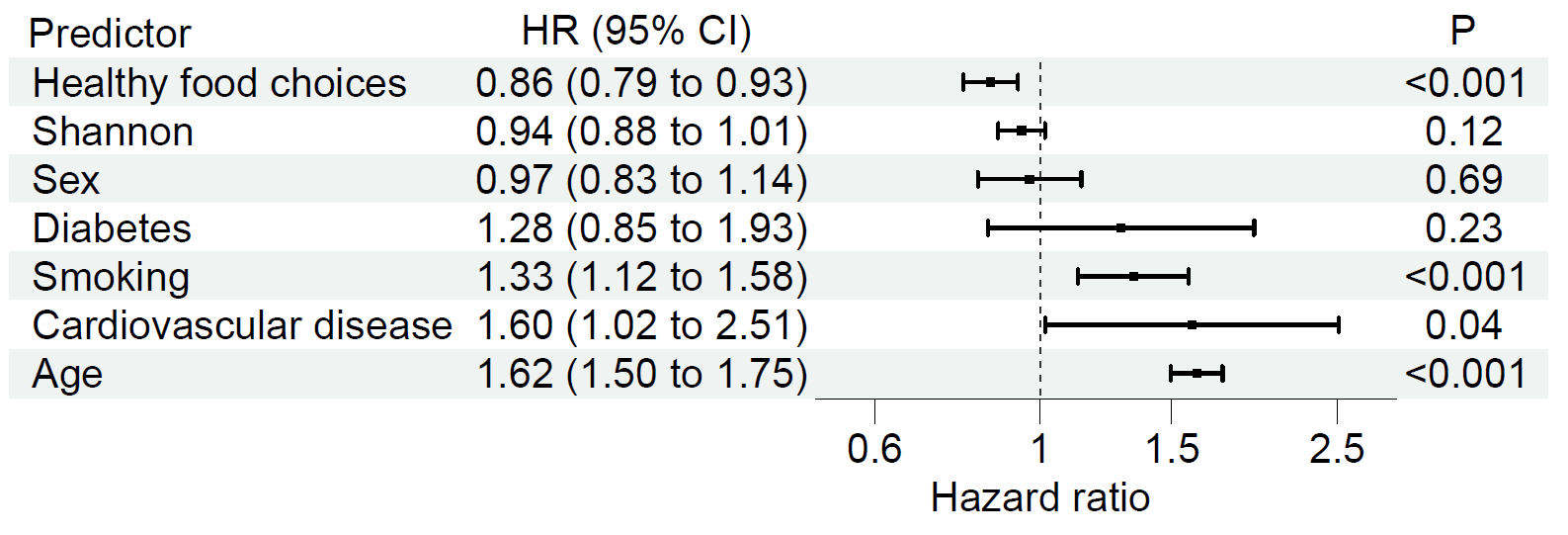


**Supplemental table S12.** Sensitivity analysis of the association between species-level relative abundance associations with incident hypertension while limiting the follow-up time to 10 years.

| Species | HR | 95% CI |  |  | FDR |
| --- | --- | --- | --- | --- | --- |
| *Allobacillus_sp007559425* | 1.16 | 1.06 - 1.27 |  |  | 0.42 |
| *AF33_28_sp003477885* | 1.37 | 1.11 - 1.70 |  |  | 0.48 |
| *Bifidobacterium_adolescentis* | 0.93 | 0.88 - 0.99 |  |  | 0.82 |
| *Enterocloster_sp000431375* | 1.15 | 1.03 - 1.29 |  |  | 0.82 |
| *Ruminococcus_C_58660_callidus* | 0.84 | 0.74 - 0.96 |  |  | 0.82 |
| *51_20_sp001917175* | 0.93 | 0.87 – 1.00 |  |  | 0.86 |
| *Akkermansia_muciniphila_D_776786* | 0.97 | 0.93 - 1.02 |  |  | 0.86 |
| *Alistipes_A_871400_communis* | 0.96 | 0.88 - 1.05 |  |  | 0.86 |
| *Alistipes_A_871400_excrementavium* | 1.07 | 0.94 - 1.21 |  |  | 0.86 |
| *Alistipes_A_871400_shahii* | 1.05 | 0.95 - 1.17 |  |  | 0.86 |

HR, hazard ratio, CI, confidence interval, FDR, false discovery rate with Benjamini-Hochberg corrected P value. Multivariable-adjusted models were adjusted for age, sex, BMI, diabetes, healthy food choices, cardiovascular disease and smoking.
